# New Findings Regarding the Effects of Selected Blue Food Colorants (Genipin, Patent Blue V, and Brilliant Blue FCF) on the Hemostatic Properties of Blood Components In Vitro

**DOI:** 10.3390/nu16131985

**Published:** 2024-06-21

**Authors:** Beata Olas, Bogdan Kontek, Natalia Sławińska, Jacek Białecki

**Affiliations:** Department of General Biochemistry, Faculty of Biology and Environmental Protection, University of Lodz, 90-236 Lodz, Poland; bogdan.kontek@biol.uni.lodz.pl (B.K.); natalia.slawinska@edu.uni.lodz.pl (N.S.); jacek.eugeniusz.bialecki@gmail.com (J.B.)

**Keywords:** blood platelet, brilliant blue FCF, blue colorant, hemostasis, genipin, patent blue V

## Abstract

Natural and synthetic colorants present in food can modulate hemostasis, which includes the coagulation process and blood platelet activation. Some colorants have cardioprotective activity as well. However, the effect of genipin (a natural blue colorant) and synthetic blue colorants (including patent blue V and brilliant blue FCF) on hemostasis is not clear. In this study, we aimed to investigate the effects of three blue colorants—genipin, patent blue V, and brilliant blue FCF—on selected parameters of hemostasis in vitro. The anti- or pro-coagulant potential was assessed in human plasma by measuring the following coagulation times: thrombin time (TT), prothrombin time (PT), and activated partial thromboplastin time (APTT). Moreover, we used the Total Thrombus formation Analysis System (T-TAS, PL-chip) to evaluate the anti-platelet potential of the colorants in whole blood. We also measured their effect on the adhesion of washed blood platelets to fibrinogen and collagen. Lastly, the cytotoxicity of the colorants against blood platelets was assessed based on the activity of extracellular lactate dehydrogenase (LDH). We observed that genipin (at all concentrations (1–200 µM)) did not have a significant effect on the coagulation times (PT, APTT, and TT). However, genipin at the highest concentration (200 µM) and patent blue V at the concentrations of 1 and 10 µM significantly prolonged the time of occlusion measured using the T-TAS, which demonstrated their anti-platelet activity. We also observed that genipin decreased the adhesion of platelets to fibrinogen and collagen. Only patent blue V and brilliant blue FCF significantly shortened the APTT (at the concentration of 10 µM) and TT (at concentrations of 1 and 10 µM), demonstrating pro-coagulant activity. These synthetic blue colorants also modulated the process of human blood platelet adhesion, stimulating the adhesion to fibrinogen and inhibiting the adhesion to collagen. The results demonstrate that genipin is not toxic. In addition, because of its ability to reduce blood platelet activation, genipin holds promise as a novel and valuable agent that improves the health of the cardiovascular system and reduces the risk of cardiovascular diseases. However, the mechanism of its anti-platelet activity remains unclear and requires further studies. Its in vivo activity and interaction with various anti-coagulant and anti-thrombotic drugs, including aspirin and its derivatives, should be examined as well.

## 1. Introduction

The prevalence of various cardiovascular diseases (CVDs) is linked with different endogenous and exogenous risk factors, including blood platelet hyperactivation [1,2]. Various anti-platelet drugs (for example, aspirin and its derivatives) are frequently used in the prophylaxis and treatment of CVDs. A report from the American Heart Association/American College of Cardiology Joint Committee on Clinical Practice Guidelines (2023) and ACC/AHA Guideline (2019) on the Primary Prevention of Cardiovascular Disease recommends (1) low-dose aspirin (75–100 mg orally daily) for the primary prevention of ASCVD among select adults from 40 to 70 years of age who are at higher ASCVD risk but not at increased bleeding risk; (2) low-dose aspirin (75–100 mg orally daily) should not be administered on a routine basis for the primary prevention of ASCVD among adults >70 years of age; (3) dual anti-platelet therapy (aspirin and clopidogrel) for 6 months post-PCI; and (4) dual anti-thrombotic therapy (DOAC and aspirin) in selected patients with CCD [3,4].

Not only anti-platelet drugs but also different dietary components (such as unsaturated fatty acids, vitamins (including vitamins A and E), and phenolic compounds) can play an important role in the prophylaxis and treatment of CVDs [5,6,7,8]. The presence of natural (e.g., anthocyanins, betanin, and curcumin) and synthetic (e.g., brilliant blue FCF) colorants in the diet can modulate hemostasis, including coagulation and blood platelet activation [6,7,9], and induce cardioprotective effects [9,10,11].

The use of synthetic and natural colorants in the food industry is regulated by different institutions, including the World Health Organization (WHO), the United States Food and Drug Administration (FDA), and others. Colorants can be classified according to their chemical structure or source. Natural colorants are most commonly derived from plant sources, such as leaves, tree bark, fruits, and roots. Synthetic colorants are more intense in color and are usually more stable [9,12]. The European Union has authorized about 43 colorants for use as food additives, while the United States has authorized approximately 30 [13]. Blue colorants are defined as organic molecules that absorb red light in the 600 nm region, which makes them appear blue to the eye. There are only two natural blue colorants on the list of food colorants approved by the FDA: grape skin extract and grape color extract [14,15]. In the European Union, four blue colorants are authorized for consumption: patent blue V (also called Food Blue 5, Acid Blue 3, Sulphan Blue, L-Blau 3, Patentblau V, C-Blau 20, Sky Blue, or C.I. 42051, E131), indigotine/indigocarmin (E132), Brilliant Blue FCF (erioglaucine disodium salt, BLUE No. 1, Acid Blue 9, E133, Alphazurine FG, FD, and C), and anthocyanins (E163) [13,16]. 

In East Asia (Japan and Korea), genipin (methyl (1S,2R,6S)-2-hydroxy-9-(hydroxymethyl)-3-oxabicyclo[4.3.0]nona-4,8-diene-5-carboxylate) from *Gardenia jasminosis* J. Ellis (also present in *Genipa americana* L.) is approved for use in food products as a natural blue colorant [17,18,19,20]. It is an iridoid derivative, which has recently gained popularity for its medical benefits (e.g., anti-inflammatory, hepatoprotective, anti-bacterial, anti-oxidative, and anti-thrombotic properties), low cytotoxicity, and usefulness as a cross-linking agent [20]. In fact, genipin’s usefulness as a blue colorant comes from its cross-linking abilities, as it can form dark blue pigments through spontaneous interactions with amino acids [21]. Genipin is used as a colorant in various food products, including beverages, nectars, juices, gels, and desserts, as well as in wool, cotton, and leather dyes. Concentrated fruit juices from *G. americana*, either alone or in combination with other fruits, are commercially utilized in the USA and the European Union [17]. The results from Fan et al. indicated that genipin can be absorbed in the intestine and transported to the liver through the portal bloodstream [22]. 

**Figure 1:**
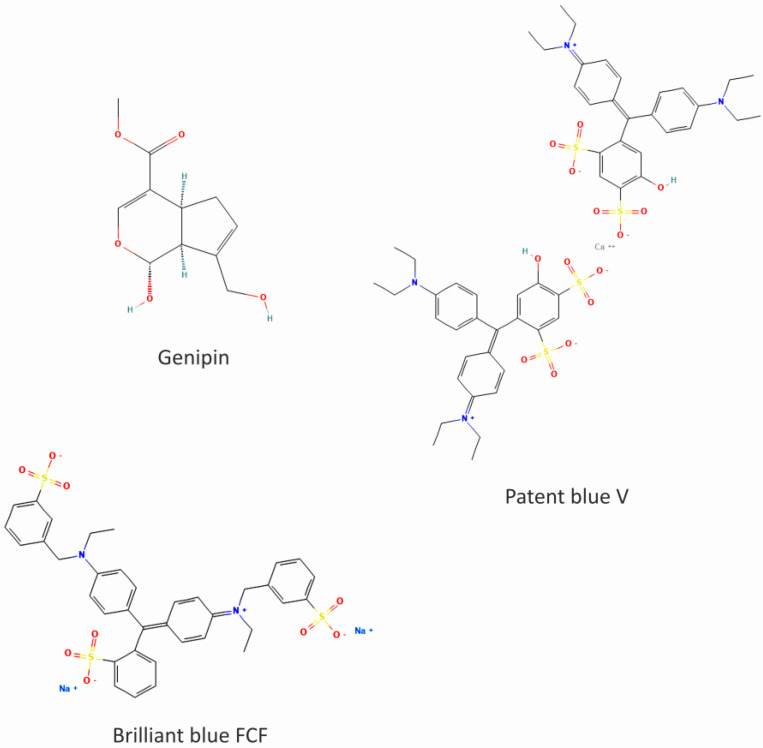
The chemical structure of genipin, patent blue V, and brilliant blue FCF. The 2D structures were obtained from PubChem (accessed on 10 June 2024). Genipin CID: 442424 [29]; patent blue V CID: 77073 [30]; brilliant blue FCF CID: 19700 [31].

However, the effect of natural (genipin) and synthetic (patent blue V and brilliant blue FCF) colorants (Figure 1) on the parameters of hemostasis has not been studied in depth. In this study, we aimed to determine the effects of these colorants on selected parameters of hemostasis in vitro. We studied their anti- or pro-coagulant potential in human plasma by measuring the following coagulation times: thrombin time (TT), prothrombin time (PT), and activated partial thromboplastin time (APTT). Moreover, we assessed their anti-platelet activity in whole blood with the Total Thrombus formation Analysis System (T-TAS, PL-chip) and in washed blood platelets through the measurement of platelet adhesion to two adhesive proteins: fibrinogen and collagen. The cytotoxicity of the colorants toward blood platelets was determined by measuring the activity of extracellular lactate dehydrogenase (LDH). We also compared the activity of the tested colorants with the activity of a commercial product, Aronox (*Aronia melanocarpa* berry extract, which has anti-platelet properties and a high level of anthocyanins) as a positive control [23,24].

The stability of genipin in aqueous solutions depends on the pH value; it degrades faster at a higher pH (e.g., 8–9) but is far more stable in neutral and acidic solutions [25]. There are no data regarding the stability of patent blue V in food, though it is stable in the dry state (it does not degrade during 5-year storage at 25 and 40 °C) [26]. Brilliant blue FCF has poor thermal stability and is susceptible to oxidation damage [26].

Importantly, the concentrations of genipin used in this study (1–200 µM) can be achieved in vivo with its supplementation. In the case of patent blue V and brilliant blue FCF, a concentration of 1 µM can be attained in blood during supplementation [26,27]. The results from Zhou et al. indicated that the recommended daily dose of genipin for adults is 6–10 g [28].

## 2. Materials and Methods

### 2.1. Chemical Reagents

Genipin and patent blue V were purchased from Pol−Aura (Morąg, Poland). Brilliant blue FCF was purchased from Warchem (Zakręt, Poland). Phosphate buffered saline (PBS), tris(hydroxymethyl)aminomethane (Tris), fibrinogen, bovine serum albumin (BSA), 4-nitrophenyl phosphate, adenosine diphosphate (ADP), collagen, and thrombin were purchased from Sigma-Aldrich (St. Louis, MO, USA). NaCl, citric acid, and sodium citrate were purchased from POCH (Avantor performance materials, Gliwice, Poland). All reagents used to measure the coagulation times were from Diagon (Budapest, Hungary). A stock solution of Aronox (*A. melanocarpa* berry extract) was purchased from Agropharm Ltd. (Tuszyn, Poland). The materials and reagents needed for the T-TAS measurements were from Bionicum Sp. z o.o., (Warsaw, Poland). The other reagents were obtained from commercial distributors and were of the highest grade available.

### 2.2. Preparation of the Stock Solution of the Tested Colorants

Genipin, patent blue V, and brilliant blue FCF were dissolved in water. The final concentrations of genipin in the blood, plasma, and blood platelet samples were 1, 2, 10, 20, and 200 μM. The final concentrations of patent blue V and brilliant blue FCF in the samples were 1 and 10 μM. Aronox was dissolved in water.

### 2.3. Blood, Plasma, and Blood Platelet Samples

Human whole blood was drawn from volunteers at the “Diagnostyka” blood collection center (Brzechwy 7A, Lodz, Poland). All volunteers (aged 25–28; *n* = 8 (5 men and 3 women)) were healthy and did not smoke. The donors did not drink alcohol or take medicine (including anti-platelet drugs, for example aspirin and its derivatives or anti-coagulants) for two weeks before blood collection. Informed consent was obtained from the participants one day prior to blood collection. The anti-coagulants were benzylsulfonyl-D-Arg-Pro-4-amidinobenzylamide (BAPA) (for T-TAS) and citrate/phosphate/dextrose/adenine (CPDA) (for other assays). The T-TAS assay was performed within 2 h of blood collection. 

All procedures were performed according to the guidelines of the Helsinki Declaration for Human Research. The research was conducted with the consent of the bioethics committee at the University of Łódź (2/KBBN-UŁ/III/2014). 

The plasma was obtained from whole blood via differential centrifugation (2800× *g*, 20 min, room temperature). The isolation of blood platelets from whole blood (via differential centrifugation) was described previously [32]. The platelet count was measured via a spectrophotometric measurement with a UV–visible Helios-α at 800 nm. Then, the platelets were diluted to the level of 2.0 × 10^8^/mL with Barber’s buffer (a modified Tyrode’s buffer: 0.14 M NaCl, 0.014 M Tris, 5 mM glucose, pH 7.4).

In all the experiments, blood, plasma, or blood platelets were incubated for 30 min at 37 °C, with either genipin (at final concentrations of 1–200 µM), patent blue V (1 and 10 μM), or brilliant blue FCF (1 and 10 μM).

### 2.4. Effect of the Tested Colorants on the Hemostasis Parameters 

#### 2.4.1. Platelet Adhesion 

Blood platelet adhesion was measured based on the activity of acid phosphatase (a platelet exoenzyme), as described previously [33]. To coat 96 well plates with adhesion proteins, 100 μL of either collagen (0.04 μg/mL) or fibrinogen (100 μg/mL) was added to the wells; then the plates were covered with parafilm and incubated at 4 °C on an orbital shaker for 24 h. The next day, the plates were washed with TBS (pH 7.5) three times; a total of 200 μL of 1% BSA was added to the wells. The plates were covered with parafilm and incubated at 37 °C for 2 h. In the meantime, to prepare the samples, the colorants were added to the blood platelets at previously described final concentrations and incubated at 37 °C for 30 min. A control sample (blood platelets with Barber’s buffer) was set up; this value was assumed to be 100%. After the incubation was complete, BSA was removed from the coated wells. The wells were washed three times with TBS (pH 7.5) with 0.1 mM CaCl_2_ and 0.1 mM MgCl_2_. Then, 100 μL of each sample was added to the wells in triplicates. In one of the plates, the adhesion of unstimulated platelets to collagen was studied; in this plate, 50 μL of TBS (pH 7.5) was added to the wells. In the rest of the plates, 50 μg of platelet agonists was added; a total of 50 μL of thrombin (final concentration—2 U/mL) was added to the wells coated with collagen and fibrinogen, and 50 μL of ADP (final concentration: 30 μM) was added to the wells coated with fibrinogen. The plates were covered with parafilm and incubated at 37 °C for 1 h. After the incubation, the wells were washed with PBS three times. Afterward, 150 μL of 0.1 M citrate buffer (pH 5.4) with 0.1% Triton X-100 and 5 mM *p*-nitrophenyl phosphate was added to the samples, and the plates were incubated at room temperature for 1 h. Afterward, 100 μL of 2 M NaOH was added, and the absorbance was read at 405 nm with the SPECTROstar Nano Microplate Reader (BMG LABTECH, Ortenberg, Germany). The data were represented as a % of the control.

#### 2.4.2. Measurement of Thrombin Time, Prothrombin Time, and Activated Partial Thromboplastin Time

Coagulation time measurements were carried out coagulometrically, according to the method described by Malinowska et al. [34]. The measurements were carried out on an Optic Coagulation Analyzer, model K-3002 (Kselmed, Grudziadz, Poland). The data were represented as a % of the control.

#### 2.4.3. Total Thrombus Formation Analysis System (T-TAS) (PL-Chip) 

The PL-chip of the T-TAS can measure the thrombus formation process in semi-physiological conditions. Full blood is pushed through a chip coated with type I collagen. The platelets are activated by collagen and 1500/s shear stress, obstructing the flow. Thrombus formation is monitored by measuring the pressure inside the flow path. Because the BAPA coagulant was used to collect the blood for this assay, secondary hemostasis was blocked, and only primary hemostasis could be studied. The results were recorded as the AUC_10_ (area under the curve), which is the area under the flow pressure curve recorded for 10 min after the test starts. The AUC_10_ depicts the growth, stability, and intensity of thrombus formation. The data were represented as a % of the control. A more detailed description of this assay can be found in Hosokawa et al. [35]. First, the colorants were added to full blood at previously described concentrations. A control sample was set up, with 0.9% NaCl added instead of the colorants. The samples were incubated at 37 °C for 30 min. Then, 320 μL of the samples was added to the PL chip, and the pressure was recorded using the T-TAS for 10 min or until the time of occlusion (when the pressure reached 60 kPa).

### 2.5. Effect of the Tested Colorants on the Parameters of Cell Damage

#### Activity of LDH

The toxic effect of genipin, patent blue V, or brilliant blue FCF on the blood platelets was analyzed via the measurement of lactate dehydrogenase (LDH) activity, according to the method described by Wróblewski et al. [36]. Blood platelets were incubated with the blue colorants at the previously described concentrations, at 37 °C for 30 min. After the incubation, the samples were centrifuged (2500 rpm, 15 min, 25 °C). The supernatant was collected. A total of 270 μL of 0.1 M phosphate buffer (pH 7.4) and 10 μL of 0.25% nicotinamide adenine dinucleotide (NADH) was added to 10 μL of the supernatant. A blank with 280 μL of phosphate buffer and 10 μL of NADH was set up. Each sample was assayed in triplicate. The samples were mixed and incubated at room temperature for 20 min. A total of 10 μL of 0.25% pyruvate was added to the samples immediately before the measurement. The absorbance was read continuously for 10 min at 1 min intervals at 340 nm. The data were represented as a % of the control.

### 2.6. Statistical Analysis

The statistical analysis was performed with GraphPad Prism 9 (version 9.5.1, Dotmatics, Boston, MA, USA). The data distribution was checked with the Shapiro–Wilk test, while the homogeneity of variance was determined with Levene’s test. Then, either a repeated measure ANOVA with Dunnett’s post-hoc test or Friedman’s ANOVA with Dunn’s post-hoc test was used. The results were presented as means ± SDs. 

## 3. Results

### 3.1. Measurement of Blood Platelet Adhesion to Collagen and Fibrinogen 

**Figure 2:**
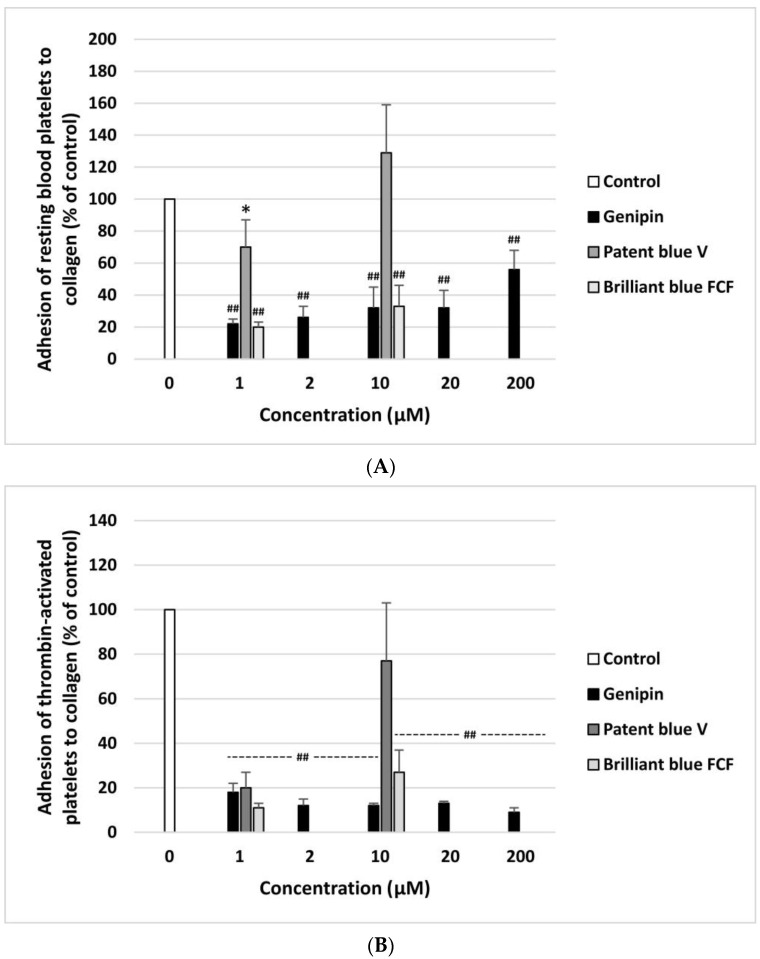
Effect of genipin (at concentrations of 1, 2, 10, 20, and 200 μM), patent blue V, and brilliant blue FCF (at concentrations of 1 and 10 μM) on the adhesion of resting platelets to collagen (**A**) and thrombin-activated platelets to collagen (**B**) (*n* = 6). In the graphs, platelet adhesion is expressed as a percentage of the control sample (blood platelets without the tested compounds). The data are expressed as means ± SDs. The results were considered significant at *p* < 0.05 (* *p* < 0.05, ## *p* < 0.001).

**Figure 3:**
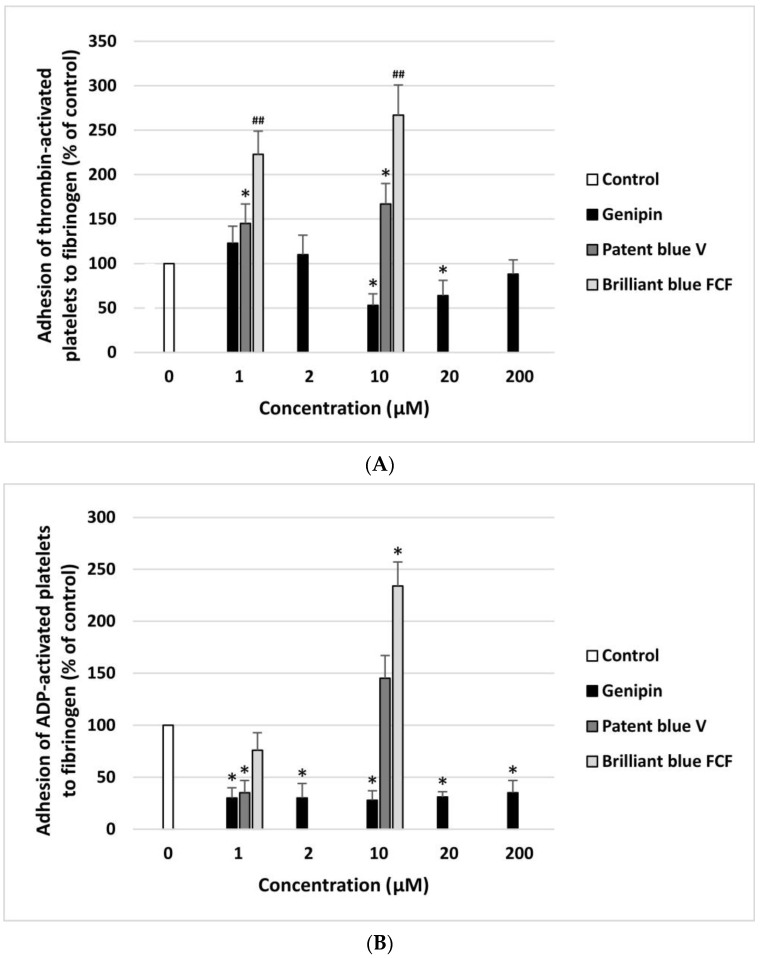
Effect of genipin (at concentrations of 1, 2, 10, 20, and 200 μM), patent blue V, and brilliant blue FCF (at concentrations of 1 and 10 μM) on the adhesion of thrombin-activated platelets to fibrinogen (**A**) and ADP-activated platelets to fibrinogen (**B**) (*n* = 6). In the graphs, platelet adhesion is expressed as a percentage of the control sample (blood platelets without the tested compounds). The data are expressed as means ± SDs. The results were considered significant at *p* < 0.05 (* *p* < 0.05, ## *p* < 0.001).

The anti- or pro-adhesive properties of genipin, patent blue V, and brilliant blue FCF are shown in Figure 2A,B. The resting blood platelets and thrombin-activated blood platelets demonstrated significantly lower adhesion to collagen after incubation with genipin at all concentrations (1–200 µM). Patent blue V and brilliant blue FCF had this effect at only one concentration, i.e., 1 µM (Figure 2A,B). Moreover, genipin significantly inhibited the adhesion of ADP-activated blood platelets to fibrinogen at all concentrations (1–200 µM) (Figure 3B). Furthermore, it significantly reduced the adhesion of thrombin-activated platelets to fibrinogen at three tested concentrations (10, 20, and 200 µM) (Figure 3A).

### 3.2. Measurement of the Coagulation Times (TT, PT, and APTT)

**Figure 4:**
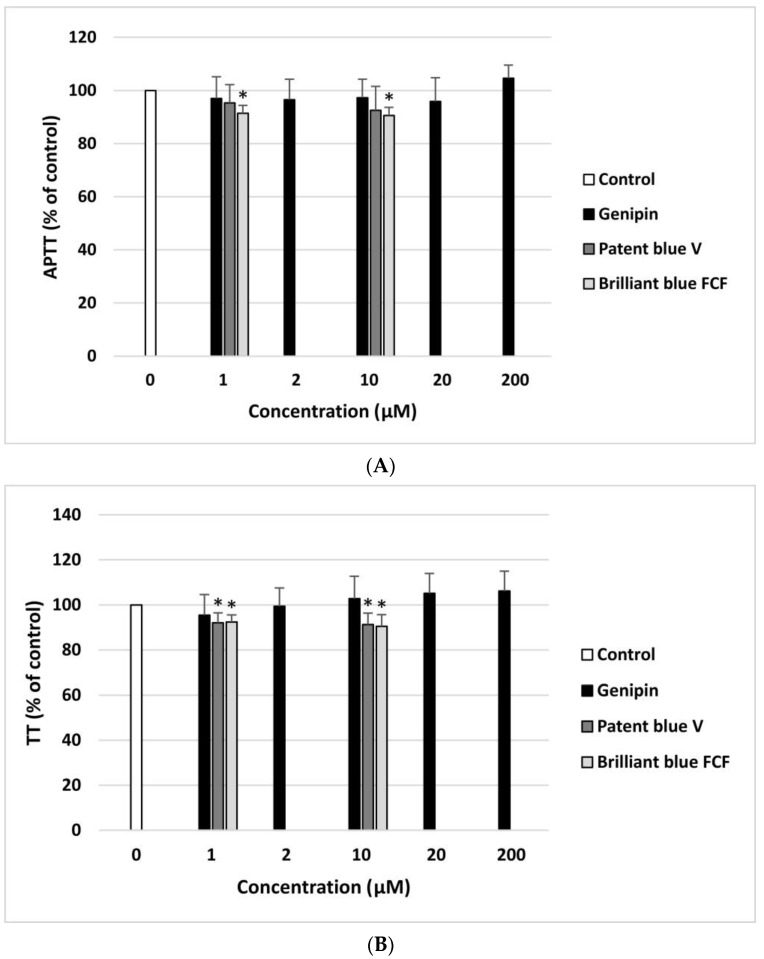
Effect of genipin (at concentrations of 1, 2, 10, 20, and 200 μM), patent blue V, and brilliant blue FCF (at concentrations of 1 and 10 μM) on the hemostatic parameters of human plasma: APTT (**A**), and TT (**B**) (*n* = 8). In the graphs, the coagulation time is expressed as a percentage of the control sample (plasma without the tested compound). The data are expressed as means ± SDs. The results were considered significant at *p* < 0.05 (* *p* < 0.05).

Genipin did not have a significant impact on the TT and APTT coagulation times measured in human plasma at any of the concentrations (1–200 µM) (Figure 4A,B). However, patent blue V (10 µM) and brilliant blue FCF (10 µM) significantly reduced the APTT (Figure 4A). In addition, these compounds (1 and 10 µM) also decreased the TT, demonstrating pro-coagulant activity (Figure 4B). On the other hand, the analysis showed that genipin, patent blue V, and brilliant blue FCF did not affect the PT at any of the concentrations.

### 3.3. Measurement of Thrombus Formation with the T-TAS (PL-Chip)

**Figure 5:**
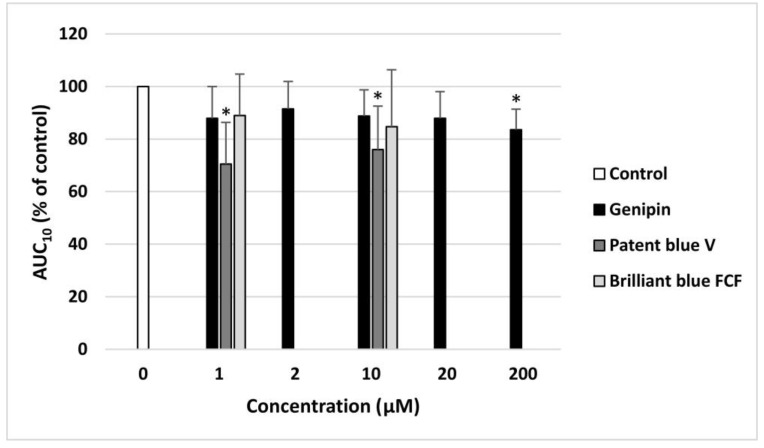
Effect of genipin (at concentrations of 1, 2, 10, 20, and 200 μM), patent blue V, and brilliant blue FCF (at concentrations of 1 and 10 μM) on thrombus formation in the whole blood (*n* = 8). The samples were analyzed with the T-TAS PL-chip at the shear stress rate of 1500/s. The results are calculated as the AUC_10_ (area under the curve). In the graphs, the AUC_10_ is expressed as a percentage of the control sample (blood without the tested compounds). The data are expressed as means ± SDs. The results were considered significant at *p* < 0.05 (* *p* < 0.05).

Genipin, at the highest concentration (200 µM), and patent blue V, at two concentrations (1 and 10 µM), significantly decreased the AUC_10_ values measured using the T-TAS in the whole blood, increasing the time needed for thrombus formation (Figure 5).

### 3.4. Measurement of Cytotoxicity

**Figure 6:**
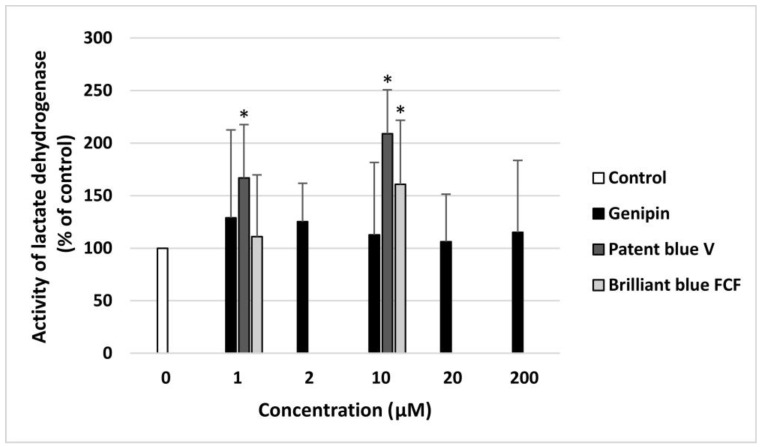
The toxic effects of genipin (at concentrations of 1, 2, 10, 20, and 200 μM), patent blue V, and brilliant blue FCF (at concentrations of 1 and 10 μM) toward human blood platelets. The results are illustrated as means ± SDs (*n* = 8). The results were considered significant at *p* < 0.05 (* *p* < 0.05).

None of the concentrations of genipin (1–200 µM) induced blood platelet lysis. However, patent blue V (at two tested concentrations: 1 and 10 µM) and brilliant blue FCF (only at the highest used concentration: 10 µM) caused statistically significant damage to human blood platelets (Figure 6).

The effects of the three used colorants on the parameters of hemostasis were compared with the aronia berry extract (5 µg/mL). The aronia berry extract, which was used as a positive control (5 µg/mL), demonstrated anti-platelet properties [23,24]. 

## 4. Discussion

The influence of natural and synthetic coloring compounds, including blue colorants such as genipin, brilliant blue FCF, and patent blue V, on the human body and the hemostatic system is a complex and not fully understood topic. Some studies confirm, and others negate the toxic properties of these compounds. Genipin possesses various potential therapeutic properties, including hepatoprotective, anti-diabetic, and anti-cancer effects [19]. It can also modulate hemostasis and affect blood platelet function [37,38]. 

The uncontrolled activation of blood platelets is one of the most important risk factors for CVDs. The presence of activated platelets in the systemic circulation is a common occurrence in various cardiovascular diseases. Research has shown that different dietary components can modulate platelet function. The results from Suzuki et al. demonstrated that genipin significantly prolonged the time required for thrombotic occlusion in an in vivo murine model. It also inhibited collagen-induced mouse blood platelet aggregation in vitro [37]. 

Genipin is a promising cross-linking agent with much lower cytotoxicity than its alternatives [20]. Liu et al. investigated the effects of a microsphere from chitosan, gelatin (Cs/Gel), and genipin (used as a cross-linking agent) on hemostasis. The results showed a significant reduction in the PT compared to the control sample. The authors suggested that Cs/Gel models the external blood coagulation cascade by shortening the time of fibrin formation and promoting the activation of the external coagulation system. In addition, Cs/Gel significantly reduced the APTT coagulation time, which may indicate that the Cs/Gel/genipin microsphere activates the coagulation cascade via the intrinsic pathway. The adhesion of blood platelets to the microsphere was also examined; at the highest concentration, genipin adhered to the lowest number of blood platelets. The authors explained that this might be due to the weakening of the positive charge of the microsphere surface by excessive amounts of genipin [39]. 

The results by Yu et al. demonstrated that a titanium nanotube biofunctionalized with a chitosan/genipin heparin hydrogel together with the controlled release of interleukin-4 accelerated the endothelization of cardiac valve prosthesis. The nanotube and the controlled release of IL-4 had a regulatory effect on the polymerization of M2 macrophages, which reduced inflammation and increased the secretion of vascular endothelial growth factor (VEGF), accelerating the endothelialization of the implant. The fast endothelialization of implant materials after valve replacement reduces platelet adhesion, which decreases the risk of thrombosis [40]. Zhang et al. also observed that genipin (20 µM) has anti-thrombotic activity in mice [38]. For the first time, using human whole blood, our data indicate that genipin at a concentration of 200 μM decreases the clot-forming ability of human blood platelets in the T-TAS PL test, indicating anti-platelet properties. Moreover, genipin exhibited anti-adhesive properties. However, more research is needed to better understand its mechanism of action, especially in an in vivo model.

Food products often include synthetic blue colorants. For example, patent blue V is used as a food colorant in the production of jellies, sauces, cheeses, dried fruits, vegetables, and liqueurs (for example, Blue Curacao). In medicine, it is used as a means of staining lymphatic vessels. In analytics, it can be utilized as a reversible indicator of redox reactions. Patent blue V is banned from use as a food colorant in the USA and Australia due to concerns about allergic reactions. However, it is allowed in food products within the European Union [26,27]. Patent blue V exhibits limited absorption after oral administration and is not metabolized. Instead, it is excreted unchanged, primarily through feces, which is its main excretion pathway. Moreover, it is selectively absorbed by the lymphatic system because it binds specifically to albumin [27]. The acceptable daily intake (ADI) of patent blue V is 0–5 mg/kg of body weight per day [26,27].

One of the most commonly used blue colorants in the food industry is brilliant blue FCF. It is used in dairy products, candies, toppings, jellies, liqueurs, breakfast cereals, chewing gums, or soft drinks [41,42,43,44,45]. The acceptable daily intake for humans has been established to be in the range of 0–12 mg/kg body weight [46]. However, a 2010 study conducted by EFSA indicated that, in people who are sensitive to this colorant, hypersensitivity reactions can be triggered by even lower doses. Brilliant blue, similarly to patent blue V, is poorly absorbed in the gastrointestinal tract. In addition, it is not metabolized in the digestive system, with approximately 95% being excreted unchanged in feces [27].

A significant and novel aspect of our findings is that a synthetic blue colorant, brilliant blue FCF, can modulate hemostasis; the experiments were carried out on human whole blood, plasma, and blood platelets in vitro. The effect of brilliant blue on metabolism, as well as its neurotoxicity, genotoxicity, and carcinogenicity, were studied in various experiments [43,47,48,49,50]. However, there is only one paper demonstrating its effect on hemostasis [51]. Molica et al. studied the function of murine blood platelets through turbidimetry after 7 min pre-incubation with brilliant blue FCF. A high dose of this compound (1 mM) completely inhibited collagen-stimulated platelet aggregation, while a lower dose (100 µM) had no effect. In addition, the researchers administered brilliant blue FCF to mice at a concentration of 100 μg/kg body weight, which resulted in an increased bleeding time relative to the control [51]. 

The measurement of coagulation times (PT, APTT, and TT) allows for the rapid assessment of different coagulation elements in vitro. PT (prothrombin time) measures the function of the extrinsic (triggered by the addition of tissue factors, calcium, and phospholipids to plasma anti-coagulated with citrate) and common (which includes factors X, V, prothrombin (factor II), and fibrinogen (factor I)) coagulation pathways. PT is usually used for monitoring patients treated with warfarin therapy, which is a vitamin K antagonist. It is also prolonged by heparin. APTT (activated partial thromboplastin time) is linked with the intrinsic (triggered by negatively charged surfaces and involving factors XII, XI, IX, and VIII, prekallikrein, and high molecular weight kininogen) and common pathways. APTT is elongated by heparin as well. TT (thrombin time) is used to assess the conversion of fibrin to fibrinogen. Adding external thrombin bypasses the intrinsic, extrinsic, and common pathways. It can be used to detect the presence of thrombin inhibitors [52]. A novel finding of our study is that this compound significantly reduced two coagulation times: TT and APTT. On the other hand, patent blue V, at the highest concentration (10 µM), only significantly reduced the APTT. A much more important pro-coagulant effect was visible when using brilliant blue FCF. Moreover, these synthetic blue colorants modulate the process of human blood platelet adhesion to adhesion proteins, stimulating the adhesion to fibrinogen and inhibiting the adhesion to collagen. The adhesion of platelets to collagen is mediated by the von Willebrand factor and GPIb receptor, while fibrinogen is bound by the active form of GPIIb/IIIa [52,53]. Interestingly, in the whole blood, only patent blue V at two concentrations (1 and 10 µM) significantly prolonged the time of occlusion, showing anti-platelet potential. The differences in the modulation of platelet adhesion observed between the colorants can be attributed to their distinct chemical structures. 

The toxicity of colorants is an important element of their biological activity assessment. The cytotoxicity of compounds intended for use in the food industry must be studied to ascertain their safety. This evaluation should be carried out using both in vivo and in vitro models. The toxicity of a natural blue colorant, genipin, has not been well-studied. Kim et al. observed that it exhibits a low level of cytotoxicity against RAW264.7, MA104, and Caco-2 cells (the viability was >90% at genipin concentrations higher than 180 µM/mL and 150 µM/mL) [54]. Moreover, there have been reports of the hepatotoxicity of genipin in rats [20]. However, in our study, none of the concentrations of genipin damaged blood platelets. These results indicate that genipin can be safe for use as a natural colorant in food products and could be used instead of synthetic colorants. According to Amchova et al., long-term exposure to patent blue V may result in a lowered hematocrit and reduced hemoglobin concentration in blood [27]. These results are in agreement with our findings. We observed that both tested concentrations (1 and 10 µM) of patent blue induced lysis in blood platelets. The other tested synthetic colorant, brilliant blue FCF, damaged platelets only at the highest concentration (10 µM). Nevertheless, there is still a need for more research concerning the cytotoxicity of genipin, especially in in vivo models [20].

## 5. Conclusions

To conclude, our results indicate that genipin, a natural blue colorant, is not toxic to human blood platelets. Moreover, because of its ability to reduce blood platelet activation, genipin could be used as a supplement that improves the health of the cardiovascular system and reduces the risk of cardiovascular diseases associated with hyperactivation of blood platelets. We examined genipin’s anti-platelet potential in two in vitro models: one based on human whole blood and the other involving human-washed blood platelets. For the first time, we showed that genipin does not modulate the coagulation system and does not increase the risk of bleeding or thrombosis in vitro. In addition, this natural food colorant might be a good alternative to synthetic food colorants, which showed pro-coagulant properties and had cytotoxic effects, especially evident in patent blue V. However, the mechanism of genipin’s anti-platelet activity remains unclear and requires further studies. Its in vivo activity and interaction with various anti-coagulant and anti-thrombotic drugs, including aspirin and its derivatives, should be examined as well.

## Data Availability

The raw data supporting the conclusions of this article will be made available by the authors on request.

## Abbreviations

ADI: acceptable daily intake; APTT: activated partial thromboplastin time; AUC: area under the curve; BSA: bovine serum albumin; CPDA: citrate/phosphate/dextrose/adenine; CVDs: cardiovascular diseases; FDA: Food and Drug Administration; LDH: lactate dehydrogenase; PBS: phosphate buffered saline; PT: prothrombin time; T-TAS: total thrombus formation analysis system; TT: thrombin time; VEGF: vascular endothelial growth factor; WHO: World Health Organization.
